# Large Out-of-Plane Displacement Bistable Electromagnetic Microswitch on a Single Wafer

**DOI:** 10.3390/s16050634

**Published:** 2016-05-05

**Authors:** Xiaodan Miao, Xuhan Dai, Yi Huang, Guifu Ding, Xiaolin Zhao

**Affiliations:** 1 College of Mechanical Engineering, Shanghai University of Engineering Science, Shanghai 201620, China; 2 National Key Laboratory of Micro/Nano Fabrication Technology, School of Electronic Information and Electrical Engineering, Shanghai Jiao Tong University, Shanghai 200240, China; xhdai@sjtu.edu.cn (X.D.); China.hy@126.com (Y.H.); Gfding@sjtu.edu.cn (G.D.); xlzhao@sjtu.edu.cn (X.Z.)

**Keywords:** MEMS, microswitch, magnetic, large displacement, microfabrication

## Abstract

This paper presents a bistable microswitch fully batch-fabricated on a single glass wafer, comprising of a microactuator, a signal transformer, a microspring and a permanent magnet. The bistable mechanism of the microswitch with large displacement of 160 μm depends on the balance of the magnetic force and elastic force. Both the magnetic force and elastic force were optimized by finite-element simulation to predict the reliable of the device. The prototype was fabricated and characterized. By utilizing thick laminated photoresist sacrificial layer, the large displacement was obtained to ensure the insulation of the microswitch. The testing results show that the microswitch realized the bistable mechanism at a 3–5 V input voltage and closed in 0.96 ms, which verified the simulation.

## 1. Introduction

MEMS (Micro-electromechanical Systems) is gradually applying widely to many applications for small-volume, batch-fabrication, and high-performance [1,2,3]. Large displacement could improve the performance of the MEMS device such as the control ability of the micro-grippers [4,5], the isolation of the microswitch [6,7], and the precision of the micro-optical switch [8,9], and so on. For RF MEMS, the large displacement could ensure effective isolation for the signal transformation for operation in the region of 10 GHz as reported in [10]. A variety of MEMS switches, including electrothermal [10], electrostatic [11], and electromagnetic [12,13,14,15,16,17,18,19,20], were reported. The electrothermal microswitch usually tends to respond slowly; as a result, its application is limited. The static microswitch is easy to fabricate, but its high driving voltage is the main obstacle for compatibility with CMOS. The electromagnetic microswitch offers a larger force and closes quickly in response at low voltage. For magnetic actuators, the large displacement is often obtained by the laterally-driven electromagnetic Lorentz force by utilizing buckling or a folded beam, and the fabrication process is based on bulk silicon processes [12,13,14]. By contrast, a vertically-actuated actuator could acquire a larger locking force than a laterally-driven actuator. However, the vertically-actuated microswitch was usually obtained by assembly processes. The electromagnetic microactuator mainly consists of the microcoil and the moving membrane, which were fabricated on different wafers, separately. Then, the permanent magnet, membrane, and microcoil were assembled by bonding or via a manual process [15,16,17,18,19,20].

This paper presented a bistable microswitch batch-fabricated on a single wafer. The process was based on surface micromachining with low temperatures and operated at low voltage, which could be highly compatibility with CMOS and solid-state devices. The different magnetic yoke type was optimized to acquire a large displacement of 160 μm to ensure the insulation characteristics of the device. Additionally, by utilizing the permanent magnet, the microswitch could remain in a latching state without an input voltage, while the power consumption decreased.

## 2. Design of the Microswitch

**Figure 1:**
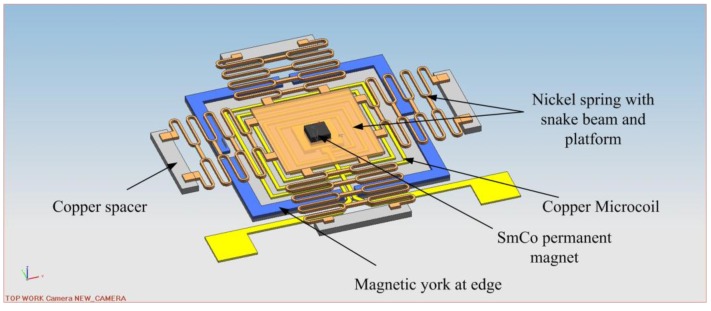
3D model of the electromagnetic bistable microswitch.

Figure 1 illustrates the architecture of the bistable microswitch. It consists of a microactuator, a signal transformer, a microspring, and a permanent magnet. The microactuator is composed of the microcoil and magnetic yoke. The microcoil is adopted to drive the actuator, and the magnetic yoke is utilized to collect the magnetic flux generated by the microcoil as fully as possible. The signal transformer comprises of a pair of contactors. The bottom contactor is on the microactuator and the top contactor is on the bottom of the microspring toward the bottom contactor. The microspring consists of a center platform and snake beams and the center platform are attracted downward or pushed upward by the deformation of the snake beams to make or break the two contactors.

There is a distance between the microactuator and microspring, suspended by the supporter, which is indicated as a displacement. When the positive voltage is fed into the coil, the positive magnetic force overcomes the elastic force, the microspring is attracted down to make the contactors, switching the external circuit “ON” (The external circuit connected with contactors). When the negative voltage is fed into the coil, the reverse magnetic force is generated. Both the magnetic force and elastic force push the microspring up to break the contactors, switching the circuit “OFF”. The permanent magnet holds the microspring in either the “down” or “up” position after switching without an input voltage, making the device a bistable microswitch. Obviously, the reliability of the microswitch depends on the balance between the magnetic force and the elastic force. As a result, the microactuator and the microspring will be designed and optimized to predict the reliability of the microswitch.

## 3. Analysis and Simulation of the Mechanical Force of the Spring

**Figure 2:**
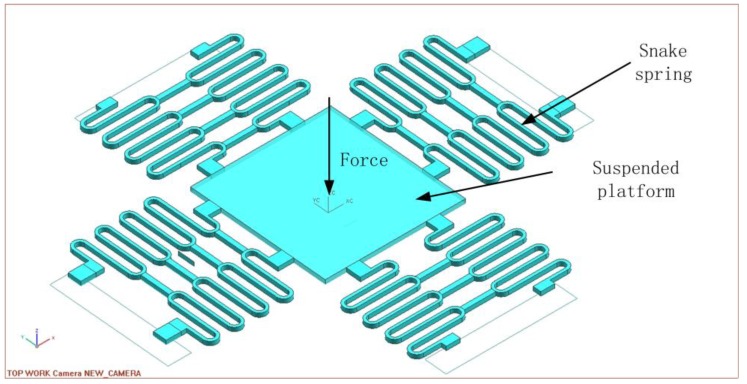
The structure of snake spring with the suspended platform.

As illustrated in Figure 2, the microspring consists of the suspended platform and snake beams. The snake beams connect with the center platform at one end and are fixed by the supporters at the other end. The center platform will move downward or upward with the deformation of the beams. Since every snake beam consists of seven straight and curving beams, the length is prolonged while the stiffness constant increases. Then, the suspended platform could sustain large displacement. At the same time, the structure parameters such the beam length L, the radius of the curving beam R, the number of curving beams n, the height b, and the width h of the beam will be optimized to maximize the displacement under the external load. Supposing the external load (electromagnetic force) is focused on the center of the platform as a concentrated force, the elastic force will be analyzed by the energy method and simulated with ANSYS 14.0 software (ANSYS, Inc., Pittsburgh, PA, USA). Then the elastic coefficiency, the displacement *versus* the given load, the frequency response, the internal stress, and the key structural parameters optimization will be completed.

### 3.1. Theory Analysis

**Figure 3:**
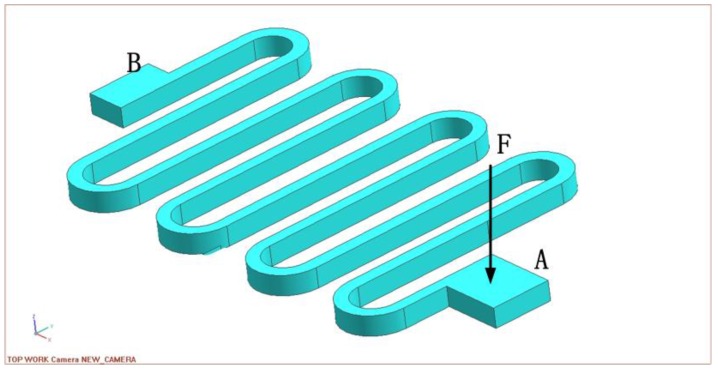
The analysis of a single beam.

The energy method is based on the mechanism that the energy produced by the external force equivalent to the power produced by the internal stress. As illustrated in Figure 3, for a single beam, supposing the external load is exerted on the point A of the snake beam, the other end B of the beam is fixed on the supporter, where the DOF in all directions is 0. The length of the straight part of the beam is L, the radius of the curving beam is R, and the number of the beams is n. The sum power is obtained by calculating the deformation resulted from the external force on every part of the beam as written in Equation (1). Then the differentiation of the power to the force is calculated, which produced the deformation as expressed in Equation (2).

According to the Castigliano’s theorem, the snake spring has:

(1) $$U=W=\sum_{i=1}^{n}\int_{i}\frac{M_{i}^{2}(x)}{2EI}dx\;+\sum_{i=1}^{n}\int_{i}\frac{T_{i}^{2}(x)}{2GI_{t}}dx$$

In Equation (1), W is the sum energy produced by the external force, which is equivalent to the U the internal energy produced by the deformation of the beams. E is the Young’s modulus, G is the shear modulus, Mi(x) is the bending moment, Ti(x) is the torsional moment, I is the geometrical moment of inertia, and $I_{t}$ is the polar inertia moment.

**Figure 4:**
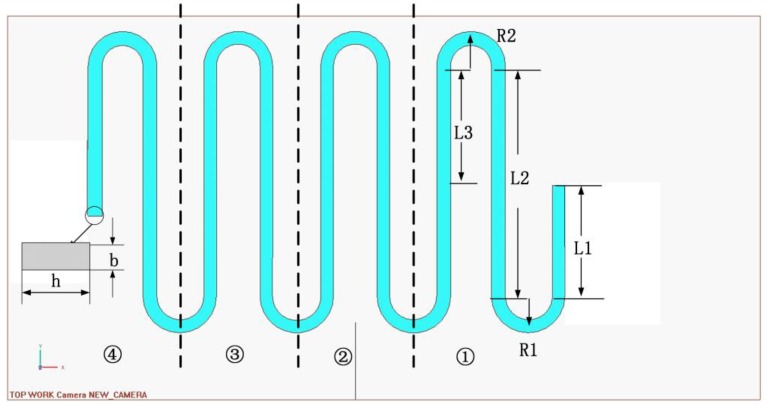
The structure and parameters of the beam.

Due to the symmetry of the structure, the single beam is selected for analysis as shown in Figure 4.

For L1:

(2) $$M_{L1}(x)=Fx$$

For R1:

(3) $$M_{R1}(x)=FR_{1}\sin\theta ,\;T_{R1}(x)=FR_{1}(1-\cos\theta )$$

For L2:

(4) $$M_{L2}(x)=Fx,\;T_{L2}(x)=2FR_{1}$$

For R2:

(5) $$M_{R2}(x)=FR_{2}\sin\theta ,\;T_{R2}(x)=FR_{2}(1-\cos\theta )$$

For L3:

(6) $$M_{L3}(x)=Fx,\;T_{L3}(x)=2F(R_{1}+R_{2})$$

(7) $$\delta_{P1}=\delta_{L1}+\delta_{R1}+\delta_{L2}+\delta_{R2}+\delta_{L3}\;=\frac{F}{EI}\left[\frac{1}{3}(L_{1}{}^{3}+L_{2}{}^{3}+L_{3}{}^{3})+\frac{3}{2}\pi (R_{1}{}^{3}+R_{2}{}^{3})\right]+\frac{F}{GI_{t}}\left[\frac{3}{2}\pi (R_{1}{}^{3}+R_{2}{}^{3})+8(R_{1}{}^{3}+R_{2}{}^{3}+3R_{1}{}^{2}R_{2}+3R_{1}R_{2}{}^{2})\right]$$

$I=\frac{bh^{3}}{12}$, b and h is the height and width of the elastic beam, where $I_{t}=\alpha h^{4}$, α is depended on the ratio between b and h, as indicated in Table 1.

The displacement of the 4 sections of beams will be calculated according to the expression, when the $\delta_{sum}$ is the displacement assumption of every part of the four beams. The D is calculated as follows:

(8) $$D=\frac{\partial U}{\partial F}=\sum_{I=1}^{4}\delta_{i}=\int_{0}^{L}\frac{M(x)}{EI}\frac{\partial (Mx)}{\partial F_{i}}dx+\int_{0}^{L}\frac{T(x)}{GI_{t}}\frac{\partial T(x)}{\partial F_{i}}dx$$

Based on Hooker’s law, the stiffness of the spring is obtained by Equation (9).

(9) $$K=\frac{F}{S}$$

### 3.2. Finite Element Analysis

The ANSYS 14.0 was used to analyze the elastic force and the displacement of the snake beam. Considering the fabrication precision, the geometric redundancy must be considered. In order to improve the efficiency of the structure, the key structure parameters will be optimized to acquire the maximum displacement with minimum external load. For a given initial value of L and R, the external load, the displacement is simulated. Then, the elastic coefficiency is obtained. According to the given maximum displacement, the corresponding external load was evaluated and exerted on the platform. Then, the internal stress distribution is acquired, which is must less than the allowable stress to avoid the mechanical failure. The material property is shown in Table 2.

**Figure 5:**
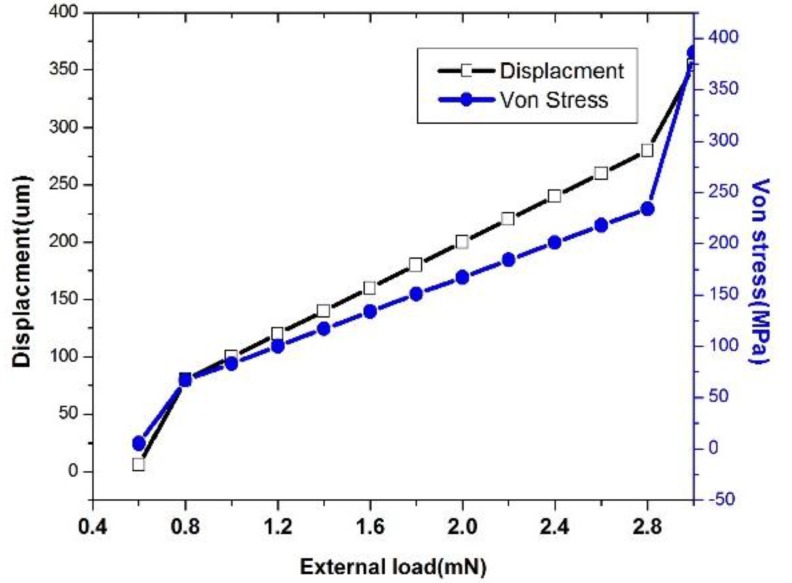
The displacement and Von stress under different external load.

The dimension of the beam is shown in Table 3. Then, the external load for the initial value is 1 mN. The external force on the suspended platform increased from 1 mN to 3 mN. Then, the corresponding displacement is simulated and shown in Figure 5. When the external load is 1 mN, the maximum displacement is 100 μm. The elastic co-efficiency is 10 N/m. When the displacement is more than 160 um, the corresponding external load is 1.6 mN. Next, the external load of 3 mN is exerted on the beam, and the maximum displacement is 354 μm. In order to avoid the mechanical failure of the device, the internal stress distribution is simulated. The maximum stress usually occurs at the connecting area of the beams and center platform. When the external load reaches 3 mN, the maximum internal stress is less than yielding stress, which indicated the reliability of the microspring.

**Figure 6:**
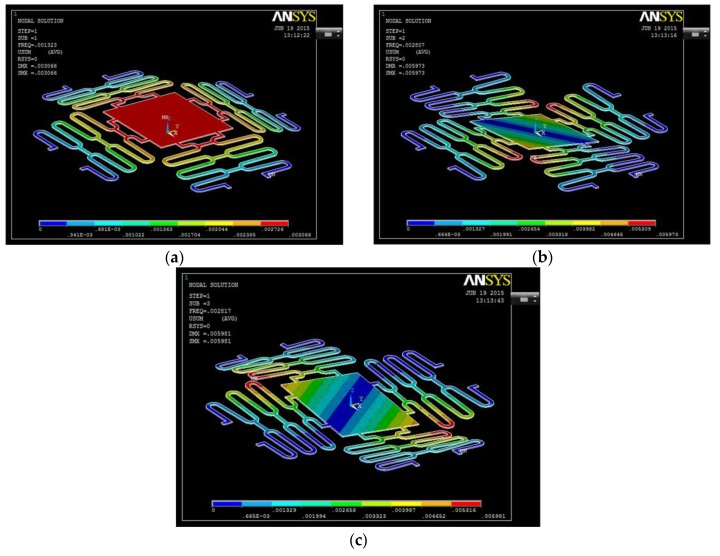
The three mode shapes of the microspring; (**a**) first mode; (**b**) second mode; and (**c**) third mode.

The model of the spring is of interest. The dynamic behavior of the moving stage is analyzed: three natural frequencies and the corresponding mode shapes. As shown in Figure 6a, the first model has a frequency of 133 Hz, and the mode shape of pure translational motion normal to moving platform. This constraint prevents the structure from being too compliant in the latter direction, which is ideal for the top electrode to contact closely with the bottom electrode. The second and third mode shapes in Figure 6b,c are rocking modes at 282 Hz along the *X* and *Y* axis. In order to avoid laterally moving of the platform, the second and third modes must be kept away. These two modes are at least 120% larger than the first mode, so the presented design provides enough separation between the desired motion and potential parasitic motions.

**Figure 7:**
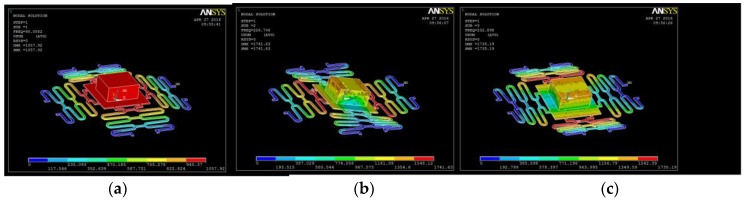
The simulation of the mode shape of the microspring with the permanent magnet. (**a**) The First mode; (**b**) The second mode; (**c**) The third mode.

In order to observe the effect of the permanent magnet on the resonant frequency of the device, the simulation of the mode shape of the microspring with the permanent magnet was carried out and described, as shown in Figure 7. According to the results, the frequency of the microspring with the permanent magnet was 111 Hz and 231 Hz. Comparing with the simulation without the permanent magnet, the resonant frequency decreased 16.5% and 14%.

## 4. Analysis and Simulation of the Magnetic Force

### 4.1. Theory Analysis

The electromagnetic force was analyzed by the segmented magnetic circuit method as presented in [21]. Considering the larger ratio between the radial length and the vertical length, the magnetic flux leakage could not be omitted. In addition, the planar microcoil with a different radius is winding around the center core, which results in the distribution of the magnetomotive force in the radial direction. In order to consider two factors mentioned above, the magnetic circuit was divided into 10 preliminary parts along the radial direction and 12 equivalent closed magnetic circuits are formed. According to the Kirchhoff’s Law, the equation for each magnetic circuit is set up. In each equation, the magnetic flux leakage is considered as magnetic resistance and the magnetomotive force is distributed into each magnetic circuit. Then the magnetic flux is solved by establishing the equation sets based on 12 equations. According to the model, the microcoil turns and magnetic yoke are optimized, which provide parameters for the ANSYS simulations for further simulation.

### 4.2. Finite Element Simulation

**Figure 8:**
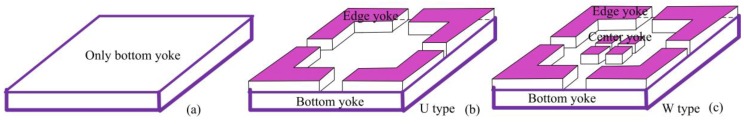
Three types of magnetic yoke designs. (**a**) Only bottom yoke; (**b**) U type yoke; (**c**) W type yoke.

The finite element simulation was used to optimize the magnetic force by simulating the different magnetic yoke type. When the current was input into the microcoil, the magnetic flux is generated and was attracted into the magnetic yoke, which attracted the upper yoke down to the bottom, then the large displacement was generated. In order to optimize the structure of the electromagnetic microactuator, three types were designed as shown in Figure 8. In Figure 8a, the microactuator consist of a bottom magnetic yoke with a planar microcoil. In Figure 8b, it consists of the center and bottom magnetic yokes. In Figure 8c, it consists of the enclosed magnetic yoke. Due to symmetry, one fourth of the microactuator was modeled and simulated.

**Figure 9:**
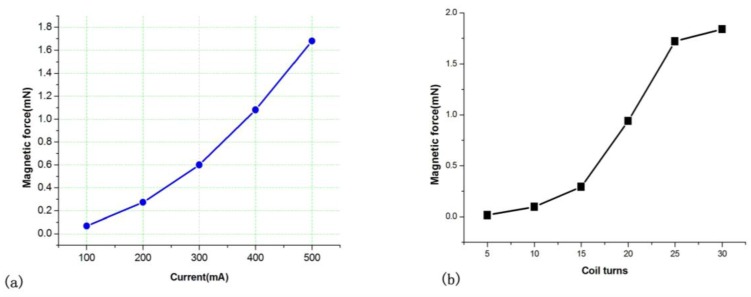
The magnetic force with different driving current and coil number turns. (**a**) magnetic force with current; (**b**) Magnetic force with coil turns.

When the input current is 500 mA, the electromagnetic force is shown in Table 4. According to the results, it is obvious that the enclosed magnetic yoke improved the efficiency of the microactuator. The magnetic force generated by the type (a), (b), and is (c) is 0.23 mN, 1.36 mN, and 1.68 mN. The magnetic force with W yoke in Figure 7c increase by 600%. The reason is that the enclosed magnetic yokes attracted the magnetic flux as fully as possible, which avoided the diffusion of the magnetic flux. At the same time, the magnetic force increased with driving current and coil number as shown in Figure 9.

## 5. Microfabrication

**Figure 10:**
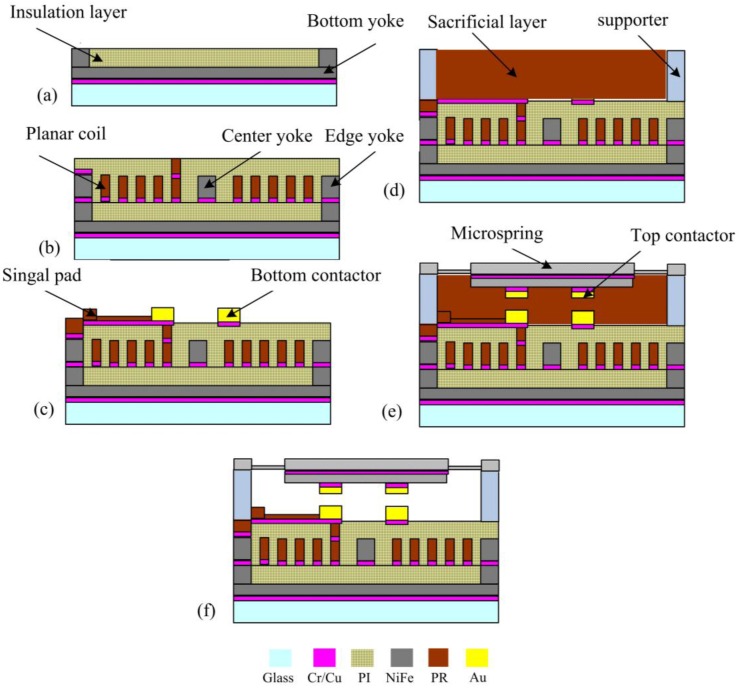
Microfabrication process of the microswitch on a single glass wafer. (**a**) bottom yoke electroplating; (**b**) coil electroplating; (**c**) bottom contactor electroplating; (**d**) supporter electroplating; (**e**) microspring electroplating; (**f**) releasing of device.

The microswitch was fabricated on an insulated substrate by electroplating technology. The large suspended air gap was achieved by the thick photoresist sacrificial layer. The main fabrication steps are sketched in Figure 10 and described as follows.

**Figure 11:**
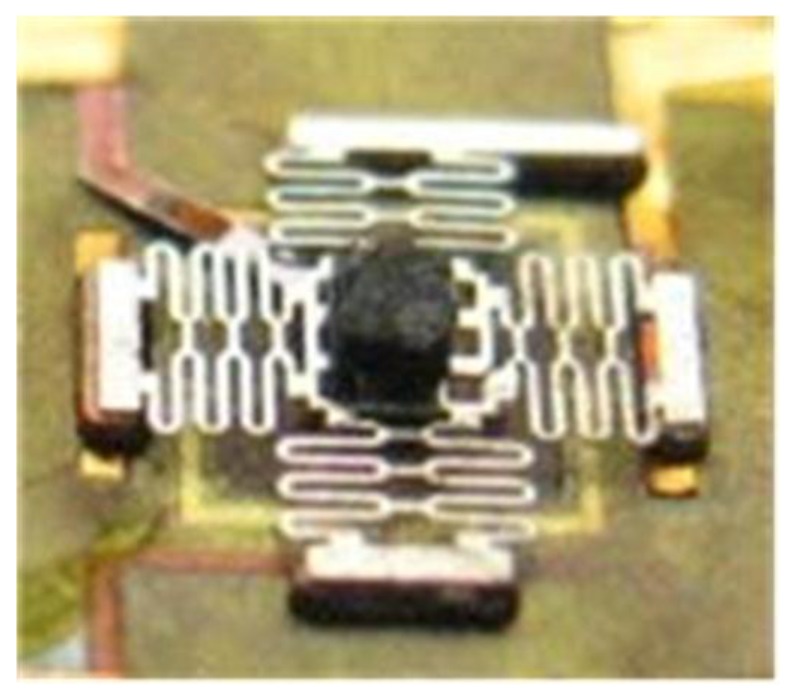
The prototype of the microswitch.

(a) Chromium/copper (Cr/Cu) was sputtered as the seed layer for plating of the device structure, then, the photoresist spin-coating and photoresist were carried out. The bottom magnetic yoke was created by electroplating the permalloy. After stripping the photoresist, the exposed seed layer was wet etched. Then, the polyimide was spin coated, thermal treated and planarized as insulation layer.
(b) After secondly sputtering the Cr/Cu seed layer and spin-coating photoresist, the microcoil, via connection and one pad was created by photolithography and electroplating copper. After stripping the photoresist and wet etching the exposed seed layer, the polyimide layer was spin coated, thermally treated, and planarized.
(c) The signal lines were created by spin-coating photoresist, photolithography, and electroplating copper after sputtering the Cr/Cu seed layer. Then the contactor on the signal line was formed by photolithography, and electroplating copper, nickel, and gold in sequence.
(d) Two layers of 80 μm thick photoresist was spin coated and thermally treated step-by-step as sacrificial material. Then the nickel supporter was electroplated with high aspect ratio structure after photolithography.
(e) The top contactors were formed by photolithography and electroplating gold, nickel, copper in sequence after sputtering Cr/Cu seed layer. Then the next Cr/Cu seed layer was sputtered, the photoresist was spin coated and the microspring was formed by photolithography, and electroplating permalloy, nickel step by step.
(f) The thick photoresist sacrificial layer underneath microspring was released in 2 wt % NaOH solution layer by layer and Cu seed layer was wet etched in ammonia solution with assistance of hydrogen peroxide and seed Cr layer was etched by an improved oxidizer etching solution. In order to avoid sticking, the wafer was immersed in ethyl alcohol for rapid evaporation to form the suspended microspring. Finally, the permanent magnet was assembled on the center of the microspring. The fabricated prototype was shown in Figure 11.

## 6. Characterization

### 6.1. Static Surface

**Figure 12:**
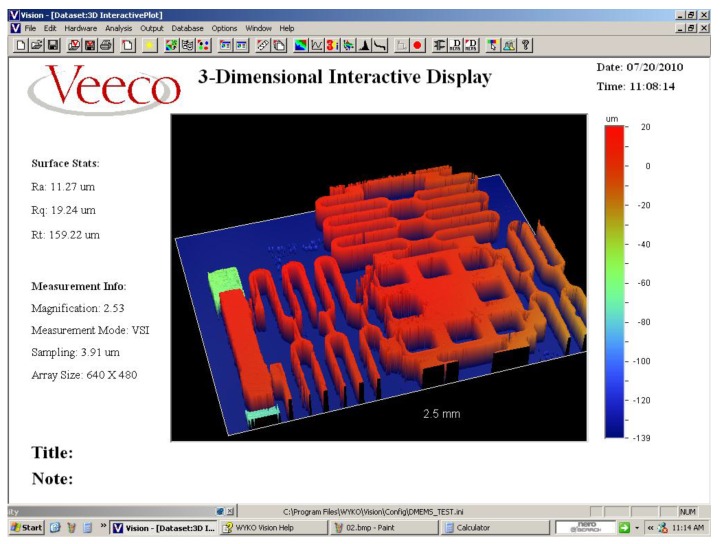
The optical profile of the electromagnetic bistable microswitch.

In order to observe the contour of the device, the Veeco 3-Dimensional Interactive optical profiler (Wyko NT9100VEECO, Veeco Instruments, Inc., New York, NY, USA) is utilized. As display in Figure 12, the Rt represent the distance between the surface of the moving platform and the substrate, which indicates the air gap/displacement, means 159.22 μm. It is means that the difference between the design and fabrication value is 0.5%, which indicates precise fabrication process.

### 6.2. Dynamic Response Testing

**Figure 13:**
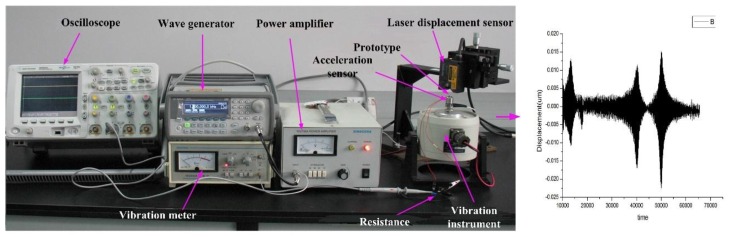
Dynamic response measurement system.

The frequency testing system is shown in Figure 13. By changing the voltage of the vibration meter, the corresponding signal is generated by a GW waveform generator (GFG-8016G, GW, Inc., Taiwan, China) incorporated with B and K 2706 power amplifier (GW, Inc., Taiwan, China). The vibration platform is activated with the prototype at the different acceleration, while the oscilloscope (Agilent MSO6034, NI, Inc., Santa Clara, CA, USA) observes the input and output amplitude. The laser displacement sensor detected the vibration amplitude at the same time and transformed it to the computer for analyzing as shown in left part of Figure 13. After a Fourier transform, the frequency information is extracted. The elastic coefficiency is obtained. The tested frequency is 125 Hz, which shows agreement with the simulation results.

### 6.3. Bistable Mechanism Test

**Figure 14:**
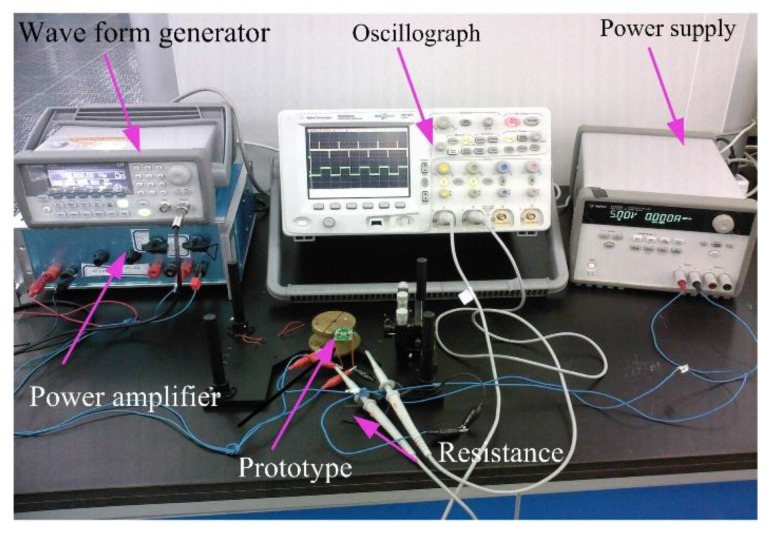
Bistabe measurement system of the prototype.

**Figure 15:**
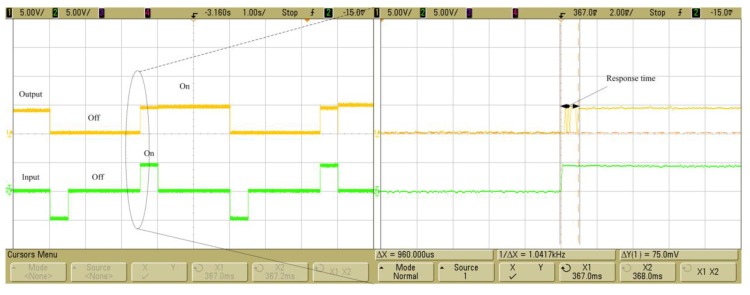
Bistable mechanism testing results and response time of the prototype.

The bistable mechanism testing system is shown in Figure 14, the 3–5 voltage generated by B and K 2706 power amplifier incorporated with a GW waveform generator (GFG-8016G, GW, Inc., Taiwan, China) was input into the microcoil, and the outoput connected with DC power supply (Agilent E3646A Dual output NI, Inc., Santa Clara, CA, USA) and a resistor. Both the input and output were monitored by the oscilloscope (Agilent MSO6034， NI, Inc., Santa Clara, CA, USA) to observe the respsonse time. The bistable mechanism testing result is shown in Figure 15, and the upper level means the input voltage while the lower level means output voltage. When the positive impulse voltage was triggered, the output voltage turned on. Then the output voltage could keep at on state without input voltage because of the permanent magnet. The bistable mechanism was realized. In order to break up the output circuit, the negative impulse voltage was triggered, and the output voltage turned off. When the current fed into the coil, the difference between the driving voltage and switching voltage at the higher level results in a response time at 0.96 ms.

## 7. Conclusions

This paper presents a microswitch fabricated on a single glass wafer, which consists of the microactuator, the signal transformer system, the microspring and the permanent magnet. Due to the thick sacrificial layer, the large displacement of 160 μm is formed based on non-silicon surface micromachining. Aiming at maximum of the elastic force and magnetic force, the structures and key parameters are optimized. Firstly, the deformation of the spring is simulated under given original parameters, then the stiffness is evaluated. The maximum displacement and corresponding stress distribution is simulated. Secondly, the microactuator structure is optimized by comparing different magnetic yoke designs. Thirdly, the frequency and stiffness of the suspended spring and the response characteristics was simulated. The measurement results agree with the simulation and show that the microswitch could work at 3–5 V and a response time is 0.96 ms.

## Figures and Tables

**Table 1:** Parameter α.

m = b/h	1.0	1.2	1.5	2.0	2.5	3.0	4.0	6.0	8.0
α	0.140	0.199	0.294	0.457	0.622	0.790	1.123	1.789	2.456

**Table 2:** The attributes of nickel.

Elastic Modulus	210 GPa
Passion ration	0.3
Density	8.96 kg/cm^3^

**Table 3:** The parameter of the microspring.

Name	Value (μm)
L1	190
L2	520
R	83
H	31
n	15

**Table 4:** The magnetic force with different magnetic yoke.

Magentic Yoke	Bottom Yoke (a)	U Yoke (b)	W Yoke (c)
Magnetic force (mN)	0.23	1.36	1.68
